# Experiences of implant loss after immediate implant‐based breast reconstruction: qualitative study

**DOI:** 10.1002/bjs5.50275

**Published:** 2020-03-17

**Authors:** B. Mahoney, E. Walklet, E. Bradley, S. Thrush, J. Skillman, L. Whisker, N. Barnes, C. Holcombe, S. Potter

**Affiliations:** ^1^ School of Psychology, College of Business, Psychology and Sport University of Worcester Worcester UK; ^2^ College of Health, Life and Environmental Sciences University of Worcester Worcester UK; ^3^ Breast Unit Worcester Royal Hospital Worcester UK; ^4^ Department of Plastic Surgery University Hospitals Coventry and Warwickshire NHS Trust, Clifford Bridge Road Coventry UK; ^5^ Nottingham Breast Institute City Hospital Nottingham UK; ^6^ Nightingale Breast Unit Manchester University NHS Foundation Trust Manchester UK; ^7^ Linda McCartney Centre Royal Liverpool and Broadgreen University Hospital Liverpool UK; ^8^ Bristol Centre for Surgical Research, Population Health Sciences, Bristol Medical School Bristol UK; ^9^ Bristol Breast Care Centre, North Bristol NHS Trust Southmead Hospital Bristol UK

## Abstract

**Background:**

Immediate implant‐based breast reconstruction (IBBR) is the most commonly performed reconstructive procedure in the UK, but almost one in ten women experience implant loss and reconstructive failure after this technique. Little is known about how implant loss impacts on patients' quality of life. The first phase of the Loss of implant Breast Reconstruction (LiBRA) study aimed to use qualitative methods to explore women's experiences of implant loss and develop recommendations to improve care.

**Methods:**

Semistructured interviews were conducted with a purposive sample of women who experienced implant loss after immediate IBBR, performed for malignancy or risk reduction across six centres. Interviews explored decision‐making regarding IBBR, and experiences of implant loss and support received. Thematic analysis was used to explore the qualitative interview data. Sampling, data collection and analysis were undertaken concurrently and iteratively until data saturation was achieved.

**Results:**

Twenty‐four women were interviewed; 19 had surgery for malignancy and five for risk reduction. The median time between implant loss and interview was 42 (range 22–74) months. Ten women had undergone secondary reconstruction, two were awaiting surgery, and 12 had declined further reconstruction. Three key themes were identified: the need for accurate information about the risks and benefits of IBBR; the need for more information about ‘early‐warning’ signs of postoperative problems, to empower women to seek help; and better support following implant loss.

**Conclusion:**

Implant loss is a devastating event for many women. Better preoperative information and support, along with holistic patient‐centred care when complications occur, may significantly improve the experience and outcome of care.

## Graphical abstract

Immediate implant‐based breast reconstruction is the most commonly performed reconstructive procedure worldwide after mastectomy for breast cancer, but up to one in ten women experience implant loss and reconstructive failure after this technique. This qualitative study explores the experiences of 24 women experiencing implant loss from six centres across the UK. It highlights the need for more balanced presentation of the risks and benefits of different techniques to inform decision‐making, the need for better information regarding the early signs of potential problems to empower women to seek help, and more support for patients experiencing this potentially devastating complication.

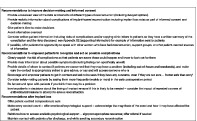

Patients need better support

## Abstract (es)

**Antecedentes:**

La reconstrucción mamaria inmediata con prótesis (*implant‐based breast reconstruction*, IBBR) es el procedimiento reconstructivo más utilizado en el Reino Unido, pero casi una de cada diez mujeres presentará pérdida de la prótesis y fallo del procedimiento reconstructivo tras esta técnica. Se sabe poco de cómo la pérdida de la prótesis afecta la calidad de vida de las pacientes. La primera fase del estudio LiBRA tuvo como objetivo explorar la percepción de las mujeres ante la pérdida de la prótesis, utilizando métodos cualitativos, y proponer una serie de medidas para mejorar la atención sanitaria de estas pacientes.

**Métodos:**

Se realizaron entrevistas semiestructuradas en una muestra de mujeres que padecieron la pérdida de la prótesis tras una IBBR inmediata, realizada por neoplasia o como procedimiento de reducción de riesgo, en seis centros. Las entrevistas analizaron la toma de decisiones con respecto a la IBBR inmediata, así como la percepción ante la pérdida del implante y el soporte recibido. Se utilizó un análisis por temas para examinar los datos de la entrevista cualitativa. El muestreo, la recopilación de datos y el análisis se realizaron de forma simultánea e iterativa hasta que se logró la saturación de datos.

**Resultados:**

Se entrevistaron 24 pacientes; 19 en las que la indicación quirúrgica fue por cáncer y 5 por reducción de riesgo. La mediana del tiempo entre la pérdida del implante y la entrevista fue de 42 (rango 22‐52) meses. Diez mujeres se habían sometido a una reconstrucción secundaria; dos estaban a la espera de la cirugía y 12 habían rechazado la reconstrucción posterior. Se identificaron tres temas clave, siendo las necesidades de: i) información precisa sobre los riesgos y beneficios de la IBBR, ii) más información sobre los signos de “alarma precoz” de las complicaciones postoperatorias que permitiesen a las mujeres buscar ayuda, y iii) mejor soporte tras la pérdida de la prótesis.

**Conclusión:**

La pérdida de una prótesis es una complicación catastrófica para muchas mujeres. Una mejor información y apoyo preoperatorios, junto con una atención holística centrada en la paciente cuando se presentan las complicaciones, podrían mejorar significativamente la experiencia y el resultado de la atención.

## Introduction

Breast cancer currently affects over 55 000 women per year in the UK^1^ and, despite improvements in treatment, up to 40 per cent require a mastectomy as the primary surgical treatment for their disease^2^. Loss of the breast may profoundly affect a woman's body image and quality of life^3^, ^4^, ^5^, ^6^, and in the UK breast reconstruction is offered routinely to improve outcomes^7^.

Immediate implant‐based breast reconstruction (IBBR) is the most commonly performed reconstructive procedure in both the UK^8^ and the USA^9^. It may offer women the benefits of a quick procedure with rapid recovery and good cosmetic results, without the morbidity associated with harvesting tissue from elsewhere^10^. Traditionally performed as a two‐stage procedure, involving tissue expansion followed by the insertion of a fixed‐volume implant^10^, the practice of IBBR has evolved significantly in recent years with the introduction of new techniques to augment the subpectoral pocket with a biological or synthetic mesh or the patient's own de‐epithelialized skin^11^. The addition of mesh creates a more ptotic, natural‐looking breast through better lower‐pole projection, and allows the procedure to be performed in a single stage^12^, ^13^, ^14^, ^15^, ^16^. These additional benefits have broadened the indications for IBBR and resulted in increasing numbers of women electing to undergo the technique^17^.

Despite the benefits of immediate IBBR, up to 10 per cent of women undergoing the procedure may experience implant loss due to complications such as infection or skin‐flap necrosis^18^. Implant loss leading to failure of the reconstruction is distressing for patients and surgeons, and has significant resource implications for healthcare providers^19^. Although rates of implant loss are often reported in the literature^20^, few studies have explored the impact of this potentially devastating complication on women's quality of life, or reported how implant loss is managed subsequently. Given the increasing numbers of women electing to undergo immediate IBBR who may potentially experience implant loss, there is an urgent need to understand women's experiences to provide patients with better information and support in the future.

Qualitative research aims to understand in depth ‘why’ and ‘how’ events arise rather than ‘how many’ occur^21^, ^22^, ^23^, and is a useful approach for exploring sensitive health issues such as implant loss. It is also suitable for conducting research that examines patient–clinician communication, how this is negotiated, and its meaning to individuals in health contexts^22^. Qualitative data can generate greater insights into individuals' perceptions of care and enable important issues to be explored by helping to develop a detailed understanding of how and why problems exist in different contexts^24^, ^25^. These data can also be used to gather information about the meanings and emotions interviewees ascribe to interpersonal processes in specific contexts that are difficult to observe through alternative methods^26^, ^27^.

The aim of this study was to use qualitative methods to explore the experiences of women who had experienced implant loss after immediate IBBR as the first phase of the LiBRA study, which aimed to use mixed methods to develop better information and support for patients experiencing this complication in the future.

## Methods

This qualitative work formed the first part of the LiBRA study. Its findings will inform the design of a questionnaire that will allow the process and outcomes of care for patients experiencing implant loss to be explored further. The study received ethical approval from the South‐West Central Ethics Committee (reference 16/SW/0115). The study was conducted in accordance with the Declaration of Helsinki. It was registered (researchregistry1002) prospectively before data collection commenced, and has been reported according to the COnsolidated criteria for REporting Qualitative research (COREQ) guidelines^28^. All patients provided written informed consent before participation in the study.

### Recruitment

Eligible patients were identified from six breast and plastic surgical units in the English National Health Service (NHS) through liaison with consultant breast and plastic surgeons, clinical nurse specialists, and review of departmental databases. Sampling was purposive to allow maximum variation of participant ages, indication for surgery (malignancy *versus* risk reduction), time from initial reconstruction to implant loss, decisions regarding subsequent reconstructive procedures (none, implant or autologous) and pathways of care (local management or referral to regional centres).

Women were eligible to take part in the study if they had undergone immediate implant‐only reconstruction following mastectomy for malignancy or risk reduction between January 2010 and December 2015, and subsequently developed infection, skin necrosis or another complication that necessitated surgical removal of the expander or implant within 9 months of the initial reconstructive surgery. Nine months was agreed by the study steering group as changes in IBBR techniques have resulted in implant loss occurring later than observed previously^29^. Women who had undergone a planned two‐stage procedure and lost their fixed‐volume implant after the second stage were also eligible. Women who had a fixed‐volume implant replaced with a tissue expander or other salvage procedure not resulting in the complete removal of the prosthesis were excluded.

An invitation letter with a reply slip and patient information sheet were sent to potential participants by the surgeon responsible for their care. Women who chose to participate were asked to return the reply slip with contact details to the study team so they could be contacted by telephone or e‐mail to arrange an interview. Sampling, data collection and analysis were conducted iteratively and concurrently as the study progressed, until data saturation was achieved.

### Data collection

Semistructured telephone interviews were conducted by a member of the research team, a female qualified chartered psychologist with no previous involvement with patients with breast cancer, using a topic guide developed by the study steering group based on a review of the literature and their collective experience as surgeons and psychologists (*Appendix* *S1*, supporting information). The interviewer was independent of the clinical teams providing patient care, and this was explained to study participants before commencing the interview. Telephone interviews are suitable for research conducted with participants who live in geographically diverse locations and, compared with interviews conducted face‐to‐face, can encourage interviewees to talk more openly about sensitive issues because they are on their ‘own turf’, with greater privacy and some degree of anonymity^30^.

The topic guide explored women's rationale for deciding to undergo immediate IBBR (primary reconstruction and, if appropriate, decision‐making for secondary reconstruction), and their perceptions of how implant loss affected them and the support they received from healthcare professionals during and after losing the implant. The topic guide was modified iteratively as the interviews progressed to allow emerging themes to be explored. Interviews were conducted at a time convenient to the study participant, and written consent was obtained before each interview. Each interview lasted for 60–90 minutes and was audio‐recorded digitally, transcribed in full, and checked by the study team for accuracy against the original recording. All transcripts were anonymized to protect patient confidentiality. No repeat interviews were performed. Field notes were made during the interviews, and taken into account during the analysis.

### Analysis

Analysis of the interviews was an ongoing iterative process, commencing soon after data collection and informing further sampling. Transcripts were analysed thematically in batches of two to four using the data analysis package NVivo (https://www.qsrinternational.com/nvivo/home), and informed by the constant comparison technique of grounded theory^31^, ^32^, ^33^. Themes drawn from the data represent distinct patterns, comprised of smaller extracts of information or ‘codes’. The analytical procedure undertaken with the first batch of transcripts was as follows: working independently, two members of the research team familiarized themselves with the transcripts in full, annotating early ideas about potential codes. Data were then analysed on a line‐by‐line basis to identify codes considered to enhance understanding of the *a priori* topic guide issues. Codes were then collated on the basis of shared, or similar, meaning in the data, and grouped into themes. Emerging themes and interpretation were presented to, and discussed with, clinical members of the study team (2 breast surgeons and 1 plastic surgeon) at several points within the study to check plausibility and triangulate findings with clinical experience. Sampling, data collection and analysis were undertaken concurrently and iteratively until data saturation was achieved and no new codes or themes were identified^32^.

Themes emerging from the study, together with examples of good clinical care described by interview participants, were used to develop recommendations to improve the experiences of patients who had implant loss in the future. Proposed strategies were discussed with the study team to determine the feasibility of implementation, explored with interview participants as the study progressed, and iteratively modified until no new potential improvements in care were identified.

## Results

Sixty‐eight women from six centres were invited to participate in the study. Of these, 28 returned a completed consent form and 24 were interviewed successfully.

Characteristics of participants are summarized in *Table* 1. The majority of women (19) underwent mastectomy for breast cancer. Participants in the risk‐reducing group were younger (median age 33 (range 25–40) years) and more likely to pursue secondary reconstruction than those having surgery for malignancy (median age 59 (range 41–74) years). Half of the total sample declined further reconstructive procedures after their experience of implant loss. Secondary reconstructions were most commonly implant‐based (8), with fewer patients (2) opting to have an autologous procedure. The median time between implant loss and study participation was 42 months (range 22–74 months).

**Table 1:** Demographics of the 24 women participating in the LiBRA study

	Total sample (*n* = 24)	Malignancy (*n* = 19)	Risk reduction (*n* = 5)
**Age (years)** *	54 (25–74)	59 (41–74)	33 (25–40)
**Length of interview (min)** *	66 (41–89)	67 (49–89)	64 (41–89)
**Time from loss of implant after primary reconstruction at interview (months)** *	42 (22–74)	48 (23–74)	41 (22–48)
**Secondary reconstruction**			
Received secondary reconstruction	10	6	4
Implant‐based	8	4	4
Flap‐based	2	2	0
Time from secondary reconstruction (months)*	37 (4–68)	38 (4–68)	37 (25–42)
Awaiting secondary reconstruction	2	1	1
Declined further reconstruction	12	12	0

* Values are median (range).

Three key themes emerged regarding women's experiences of IBBR and implant loss. These were broadly similar irrespective of the indication for surgery: having realistic, accurate information about the risks and benefits of implant‐based reconstruction to allow patients to make informed decisions about reconstructive surgery; knowing the ‘early warning’ signs of postoperative problems to empower patients to seek help; and the need for ongoing support after implant loss.

### The need for realistic, accurate information about risks and benefits of implant‐based reconstruction to inform decision‐making

All women described how their surgeons presented implant‐based reconstruction more positively than other forms of reconstruction in preoperative consultations, and that this influenced their decision to opt for the procedure.

*‘I just didn't want to be lying about … you're in hospital a lot longer with the back flap… recovery time is much worse … The mastectomy, followed by the implant, was the quickest way I could get back on my feet’*

Patients' focus on a procedure that offered fast recovery was motivated by their desire to regain a sense of physical, psychological and social normality, and these factors were central to their decision to opt for IBBR.

*‘… you know I wanted to look normal … I wanted to feel like a woman’*

Women who were considering surgery following a new breast cancer diagnosis also reported a sense of urgency about the decision.

*‘They want you to do everything quickly … from diagnosis to surgery, I think there were some number of weeks … that they needed to comply with … there was a sort of urgency about it.’*

This often resulted in women making quick decisions about surgery based on what was presented as the ‘easiest’ and ‘quickest’ option by their surgical team. On reflection, some women felt they perhaps should have explored their options more fully, but did not feel able to do so at the time.

*‘If somebody's talking statistics, I'm probably just thinking, “Actually, I want my cancer out. Can you just fix me?” If anybody had said to me at that time, “We can fix your cancer, but we need to chop your left arm off,” I'd have probably just said, “Do it” … your whole sense of normality is huge. You're fighting for your life. You've been told you've got cancer’*

Women's decisions for implant reconstruction were particularly influenced by their surgeon's optimism that the procedure would be successful.

*‘… the surgeon was very optimistic … so I don't think I explored … any other issues that might ensue’*

Some women could not recall the risk of implant loss and reconstructive failure being discussed.

*‘I suppose I didn't realise the number of [implant losses], I'm not sure whether they know that, or they're supposed to tell you that. I didn't know how many implants actually failed’*

Others described discussing potential risks, but explained that their surgeon explored this with them, and they felt reassured.

*‘There's risk involved in anything, however this … doesn't look too bad, so I'll go for it … It was just that positiveness … I had every right to be really sure of (my surgeon)’*

Although women undergoing risk‐reducing surgery did not perceive the same urgency regarding decision‐making for surgery, they – and some patients undergoing surgery for breast cancer – described how it was difficult to conceptualize risk and what that meant for them.

*‘… like many people before you go into something … you assume you're not going to be one of those people … it's very hard to quantify what that means and how likely something is’*

Optimism bias, however, emerged as a common theme, irrespective of indication for surgery.

*‘… never looked at the negative side of it … I thought it will work’*

One of the reasons for this optimism was that the surgeons often appeared to downplay the risk of complications associated with IBBR. Some women described how they would have valued more information about the actual risks of surgery rather than the general, almost paternalistic, reassurance they received.

*‘I definitely feel like I would have appreciated more information about … the risks … The probability of these things happening rather than just … these things probably won't happen to you’*

Indeed, the majority of patients felt that the provision of more realistic information about the potential risks associated with IBBR could have enabled them to make a more informed decision about surgery, even if their ultimate decision did not change.

*‘1 in 10, see I don't know whether I was aware of that. I don't remember those figures … Yeah. I don't remember them saying too much about the risk. I think if I'd known about that 1 in 10, perhaps I would have thought about it a bit more. That's quite a lot isn't it?’*

Full and frank discussion of the magnitude of the risks involved was considered to be a very important, though often lacking, component of the decision‐making process. Even patients undergoing IBBR for risk reduction, who had more time to meet with their surgeon and discuss their options, reported how discussions around the risks associated with surgery did not occur.

*‘Obviously, maybe if I had been given more information on the after bit I might have been more prepared possibly … I wasn't a smoker. I wasn't overweight. I was generally quite healthy. I've got thyroid problems but that's all under control. Yes, I thought I was healthy. I was in my early 30s. I thought those risks would be more likely if you were in your 40s, overweight and a smoker. You know?’*

Although accurate information about the magnitude of the risks may not have changed many women's decision to have IBBR, it may have minimized their belief that they were to blame for the loss of the implant.

*‘… it's my body, it's my fault that I've lost them … I've only ever met one person that's lost their implants and I didn't realise it was 1 in 10’*

Better information provision may also have minimized the guilt that some women described as they blamed themselves for not asking the right questions before surgery.

*‘I've got to take some responsibility myself … I should have asked more questions, but it didn't occur to me, and I'm from a generation that, you know, doesn't want to take up people's time, so I don't want to be too questioning when I'm with people who are incredibly busy’*

In addition to more information from their surgical teams, many women highlighted how much they felt they would have benefited from other sources of information, in particular meeting other women who had undergone IBBR and alternative reconstructive procedures, with both successful and less successful outcomes.

*‘And maybe … say groups … meetings or whatever with other people in the same situation … I think you get a lot out of that when you're just talking to other people’*

### The need to empower patients to identify and act on potential problems early

In addition to not fully understanding the likelihood of experiencing a postoperative complication, the majority of women felt they had not been given sufficient information to identify and respond confidently to signs of complications with their implant.

A number of women described being uncertain about how to interpret their concerns about their implant because they did not expect problems to arise, given that clinicians had emphasized the success rates of IBBR. The quality of information provided at discharge was described by a number of women as making it difficult for them to identify and act on problems with the implant.

*‘I had all that information but nothing I would say specific about caring for the wound (except) … I had a dressing and it wasn't coming off’*

Some women reported feeling unwell after surgery, but not appreciating that this may represent a problem, such as infection, with their reconstruction.

*‘I just thought, oh, I've had an operation, I'm not taking very well’*

A number of women who did have concerns about their implant described how they had found it difficult to access help or to know who to ask in response to their concerns. Some were dismissed.

*‘… as I say, this is my first experience of being a patient, I wasn't very bolshy and confident, and I didn't want to bother people, so I was ringing up the breast care nurses saying: “Who do I talk to?” – the breast care nurses said: “Oh, your breast care nurse is on maternity leave, I can't really help you … and basically told me to go away’*

Other patients did not feel confident about raising their concerns about their implant with clinicians.

*‘I remember thinking, “It's leaking. I'm not quite sure what I need to do about this”, which is why I rang the doctors … I think if the hospital had talked to me about what to do about that, I wouldn't have then thought to myself, “Okay, what do I do about this now?”’*

Uncertainty about how to identify problems with their implant was related to feelings of self‐blame by a number of patients.

*‘I just thought: “What have I done wrong? Have I done something wrong?” … I thought, no … I've been very careful … I think you do blame yourself’*

Once a problem had developed, some women described being seen by someone without specialist knowledge, such a member of the on‐call surgical team whom they felt, in retrospect, did not manage them appropriately, and that this may have influenced the outcome.

*‘Over the weekend, underneath my left breast started to look a bit red along one of the surgical wounds … So, I came to the ward at the weekend, but I think it felt like, perhaps, they weren't particularly well set up for it, because it was the general plastics registrar that I saw … He had a look … and said, “No, it's probably fine. They'll see you next week”. Knowing again what I know now, having been through everything, I think I would probably have asked him to call my surgeon, because I think I would have started antibiotics then if it had been one of the breast surgeons …’*

For some women, the infections that ultimately led to implant loss went on for a protracted period of time, with a significant impact on their psychological well‐being.

*‘I couldn't talk without crying … lived 12 months on antibiotics … always in hospital … I got myself into a very low place’*

Other women described how some clinicians were reluctant to make decisions, especially regarding implant removal.

*‘I wasn't really happy just waiting to decide whether to have it taken out … I got the impression nobody wanted to make a decision’*

In addition to more information about possible signs of complications and better signposting to points of contact, a number of women felt that, in retrospect, being seen earlier in the postoperative period may have allowed their problems to be detected sooner.

*‘… it might have been appropriate to check the dressing … after about a week or so rather than two weeks. I'm not a medical person … but when I became aware that something wasn't quite right … I did have to wait a couple of days or so before it actually got sorted’*

### The need to offer more support to patients after implant loss

Psychological acceptance of losing their implant was reported by some women. However, the majority felt implant loss had affected them negatively, psychologically and socially, and the lack of support available to them during this time aggravated these adverse consequences.

*‘Psychologically … like a new bereavement … I'm back to square one again. I do feel quite low …’*

Even patients who felt well supported by family and friends still reported sustained psychological distress following implant loss.

*‘Although I have got a very close family, I've, sort of, lost – not lost the meaning of life; I've, sort of, lost all my motivation to do anything good’*

Many women described how implant loss impacted on their body image.

*‘… it was just looking down at it … And then when I looked in the mirror, you know it was just horrible’*

Others described how losing an implant affected their lifestyle, and in some cases their livelihood.

*‘… it cost me my job … I couldn't go back to work. I couldn't deal with it mentally. Physically, I can't stand up properly’*

For women who disclosed a pre‐existing mental health problem, losing their implant was perceived to have led to a recurrence of the condition.

*‘I have had depression and that actually did come back’*

Some women blamed themselves for the complications they experienced, and others described regretting their decision to have implant‐based reconstruction.

*‘… looking back, it wasn't a good decision, and when I'd had the implant, just for those few days that I had the implant, I knew it was wrong for me’ ‘I think, oh it's my body, it's my fault that I've lost them’*.

Although implant loss was described as affecting every aspect of the women's lives and was associated with significant psychological distress, the majority of women found little support available to them once their implant had been lost, with little or no contact initiated by clinical teams. This led to some women feeling abandoned.

*‘(after implant removed): They shook our hand and said, “We'll see you next year” … and I went, what do I do now? I've come out of hospital yesterday … I didn't know what to do … I just felt cast out … there wasn't really any support there at all’*

Specialist psychological support was perceived as needed by the majority of patients after implant loss, but for many this was missing from the care they were provided with.

*‘I think it would have been useful to maybe been offered for somebody to speak to, you know like some sort of like psychological type of support’*

A number of patients felt that a more empathic and patient‐centred approach was needed from clinicians managing patients who had experienced implant loss, especially when discussing further reconstructive procedures.

*‘… for the surgeon, a little tweak is not very much – for a patient that's a whole … another waiting for a date … time off work … being under anaesthetic, which is just as significant almost as your initial operation … that's part of what needs to be up front … made a lot more clear … how likely it is that you know you'll need another operation’*

For women electing to undergo further reconstructive procedures, the uncertainty around waiting lists and further surgery was also perceived as an additional stressor.

*‘…living with uncertainty is very very uncomfortable … when things go wrong and you're having multiple surgeries and you're having to do that again and again … it was probably two years by time and I had everything sorted – that's a long time to live with the anxiety of not knowing what's going to happen next’*

This led to patients feeling dismissed or abandoned by their surgeon after implant loss.

*‘… I don't feel like there was any support given from the hospital itself, never got offered any … even if it was a phone call or a letter just to stay that there's still a waiting list or something like that, instead of me feeling, and I still feel like that, that they just forgot about it’*

One participant summarized the improvements that are needed when managing patients who have experienced implant loss, highlighting the need for compassionate patient‐centred care.

*‘… perhaps this will highlight that through the whole patient journey the healthcare experts involved need to make every contact count in respect to emotions and coping and offer … emotional and psychological support’*

Recommendations for improving the experiences of patients undergoing IBBR and implant loss on the basis of these data are summarized in *Table* 2.

**Table 2:** Recommendations for clinicians working with women undergoing implant‐based breast reconstruction

**Recommendations to improve decision‐making and informed consent**
Provide a balanced view of the risks and benefits of different types of reconstruction (including delayed options)
Provide realistic information about complications of implant‐based reconstruction including implant loss rates as part of informed consent and decision‐making
Give patients time to make decisions
Avoid information overload
Consider written patient information including risks of complications and/or copying clinic letters to patients so they have a written summary of the consultation and the risks discussed; see *Appendix* *S2* (supporting information) for example of information sent to patients
If possible, offer patients the opportunity to speak with other women who have had reconstruction, support groups, or other patient‐centred sources of information
**Recommendations to empower patients to recognize and act on possible complications**
Clearly explain the risk of complications so that patients are aware these could happen and know to look out for them
Provide clear information about possible symptoms (including feeling non‐specifically unwell)
Provide details of whom to contact if patients are concerned that they may have a problem (**including out‐of‐hours and weekends**), and make sure the contact is appropriately skilled to give advice, or can and will access someone who is
Encourage and empower patients to get in contact and ask to be seen if they have any concerns, even if they are not sure – ‘better safe than sorry’
Consider safety‐netting patients by seeing them more frequently (weekly or more) in the early postoperative period
Be honest and open with patients if you think there may be a problem
Involve patients in decisions about the timing of implant removal if this is likely to be needed – consider the impact of repeated courses of antibiotics/admissions to attempt to save a reconstruction
**Recommendations after implant loss**
Offer patient‐centred compassionate care
Make every contact count – offer emotional/psychological support – acknowledge the magnitude of the event and how it may have affected the patient
Reinforce how to access available psychological support – signpost appropriate resources; offer referral if needed
Maintain contact with patients after discharge, and while awaiting secondary reconstruction

## Discussion

Implant loss will affect almost one in ten women undergoing implant‐based reconstruction in the UK^18^, but this is the first study to explore women's experiences of implant loss and how it impacts their quality of life. Key findings were similar, irrespective of the indication for mastectomy. They include: the need for accurate and balanced presentation of the risks and benefits of implant‐based procedures to allow women to make fully informed decisions about surgery; empowering women to recognize and act on early warning signs of implant‐related problems; and the need to support women clinically and psychologically if they need to have their implant removed following a postoperative complication. A more balanced discussion, including potential risks, may actually decrease the number of implant‐based procedures performed, as a number of women in the study, particularly those having surgery for malignancy, elected to undergo reconstruction only because it was presented as a ‘quick and easy’ option. Many of the issues identified could be addressed by better communication, appropriate signposting, and focusing on delivering compassionate, patient‐centred care – ‘making every contact count’. Implementing the simple recommendations from this study into clinical practice would be a major step towards significantly improving the experience of implant loss for women who develop this potentially devastating complication.

Implant loss is likely to become a major problem worldwide as rates of implant‐based breast reconstruction continue to increase^9^, ^17^. The growing magnitude of the problem is reflected in the numbers of recent studies reporting the clinical outcomes of autologous^34^, ^35^, ^36^, ^37^, ^38^, ^39^, ^40^ or further implant‐based^41^, ^42^ reconstruction performed after a failed implant‐based procedure. Although the technical success rates of further reconstructive surgery are high, few studies have included patient‐reported outcomes (PROs) in their assessment of success^43^. The value of existing PRO questionnaires in this group, however, is questionable, as previous questionnaire‐based breast reconstruction studies^44^, ^45^ have suggested that complications, including implant loss, do not adversely affect women's long‐term quality of life, in stark contrast to the present findings. This may be because patients with complications such as reconstructive failure do not self‐identify as having had a reconstruction, so elect not to participate; a number of additional reasons for response bias in breast reconstruction studies have been reported recently^46^. The sensitivity of existing PRO measures, however, is an important consideration for future research, as many of the themes identified in the present study, in particular the perceived lack of support following reconstruction failure, clearly resonate with those identified in a similar qualitative study^47^ that explored the impact of flap loss on patients undergoing autologous reconstruction. The differences in the themes identified between the two qualitative studies reflect key differences in the procedures performed and timing of postoperative complications, in particular that flap failure occurs while patients are still in hospital. This means that patients undergoing autologous reconstruction are not required to recognize the signs of potential problems or know when to seek help – a key finding in the present study. The differences in satisfaction with information between breast surgeons (largely performing implant‐based reconstruction in the UK) and plastic surgeons performing free‐flap procedures has been reported previously^48^. These rich, clinically meaningful data suggest that qualitative approaches may be a more appropriate method for exploring this difficult and sensitive issue, and better quantitative methods will be needed for use in future studies.

This study has highlighted several areas for improvements in practice, but it has limitations. First, the recruitment rate was low. Sixty‐eight women from six centres were invited to participate, but only 24 were interviewed successfully. There is, therefore, likely to be response bias in the study. It is not clear, however, whether the women who elected not to participate did so because they were more significantly affected by their implant loss than those included, or whether they experienced better outcomes and moved on with their lives. Further work in a wider group is therefore needed to explore experiences of implant loss. Recall bias, particularly with regard to information provided about reconstruction, is also a possibility, given the duration of time between implant loss and interview (median 42 months). All participants, however, clearly described how implant reconstruction was presented as a straightforward procedure; irrespective of whether specific details were discussed, this potentially inaccurate representation of implant reconstruction had particular salience for the women in the study, and is therefore likely to be an accurate representation of their experiences. In addition, although every effort was made to sample study participants purposively, fewer patients undergoing risk‐reducing surgery and few patients who went on to have autologous reconstruction were included in the present study. Again, the reasons for this are unclear. It would be helpful to compare the characteristics of those who agreed to participate with those who did not, but ethical approvals did not permit data collection on non‐consenting patients, precluding this analysis. Finally, the experiences of this limited cohort may not be generalizable to women experiencing implant loss in other centres across the UK. Participants, however, were sampled purposively with respect to age, indication for surgery and secondary reconstruction from six centres at geographically diverse locations, and had been treated by multiple surgeons at both local hospitals and regional referral centres, suggesting the inclusion of a breadth of perspectives and experiences. Interviews were continued until data saturation was achieved, so it is unlikely that additional participants from other centres would have identified significant further themes. Furthermore, the patient experiences were triangulated with those of surgeons and specialist nurses, both within the study team and through presentation of key findings at an Association of Breast Surgery annual conference. Feedback from meeting attendees, both during the question‐and‐answer session and informally after the presentation, confirmed the findings, supporting the robustness of the present study^49^.

This qualitative work is the first phase of the LiBRA study, which aims to use mixed methods to explore the experiences of women who undergo implant loss after immediate IBBR to inform the development of better information and support for those who experience this complication in the future. The simple recommendations proposed based on this initial work represent significant progress towards achieving this aim. Further work is now needed to develop a questionnaire to explore the outcomes of implant loss in a broader group of patients, to determine the extent to which the issues identified in the present study are widespread and to identify additional areas where improvement is required. Recognizing areas of good practice will also be a key element of the questionnaire study, as these will form the basis of further recommendations for information and support. Effective strategies to optimize communication of risk, share information to empower women to promote the early identification of complications, and encourage compassionate patient‐centred care for women who experience problems will then be needed to improve outcomes for patients experiencing implant loss in the future. Ultimately, however, rates of implant loss in the UK^18^ are unacceptably high compared with current best practice guidelines^50^. Research to identify evidence‐based best practice for IBBR^18^, ^51^ and reduce implant losses will therefore be the most important strategy to improve outcomes meaningfully for patients electing to undergo implant‐based procedures in the future.

## Supporting information


**Appendix S1.** Supporting information
