# Supplementary material for: Experiences of implant loss after immediate implant‐based breast reconstruction: qualitative study
Source: BJS Open. 2020 Mar 17;4(3):380–90. doi: 10.1002/bjs5.50275 (PMC7260419; doi:10.1002/bjs5.50275)
Supplement: Supplementary file 1 — Appendix S1. Supporting information [file BJS5-4-380-s001.docx]

**BJS5_50275**

**Experiences of implant loss after immediate implant-based breast reconstruction: qualitative study**

**B. Mahoney, E. Walklet, E. Bradley, S. Thrush, J. Skillman, L. Whisker, N. Barnes, C. Holcombe and S. Potter**

Appendix S1 Topic guide for semistructured interviews

**Introduction**

- *Introduce self and research question*
- *Explain need to record interview and test audio recorder*
- *Written consent obtained prior to interview – verbally confirm consent to interview and audio recording*

**Clarification of details and background information**

- *Name and age*
- *Indication – malignancy (invasive/DCIS) or risk reducing*
- *Any adjuvant treatment (Chemotherapy/radiotherapy)*
- *Centre of primary reconstruction*
- *Date of primary reconstruction*

**Primary reconstruction: timeline, course of management and decision making**

- *Could you talk me through what happened before your (first) reconstruction?*
- *How did you come to the decision to have your (first) reconstruction?*
- *What happened after your (first) reconstruction?*

**Primary reconstruction: experiences of implant loss**

- *Could we now talk about your experiences of implant loss – how did you feel when this happened?*

**Primary reconstruction implant loss: timeline, course of management and decision making**

- *What happened next after your implant had to be removed?*
- *After you had made that decision (either secondary reconstruction or no secondary reconstruction), what happened next?*

**Psychology effects of implant loss**

- *Could we now talk about how your experiences have affected your life – how has the experience of breast cancer affected your life?*
- *How has the experience of implant loss affected your life?*
- *How has the care given to you during implant loss affected your life?*
- *How has the experience of secondary reconstruction affected your life (if appropriate)?*

**Support from Breast Unit**

- *What sources of support did you use?*
- *Which of these sources of support were the most helpful*
- *What was particularly helpful about the support received from the Breast Unit and/or Plastic Surgery Unit?*
- *Was there anything particularly unhelpful about the support received from the Breast Unit?*
- *Overall how could the Breast Unit best support the needs of women who have experienced implant loss?*

*Finally is there anything else you would like to add about your experiences of implant loss?*

Thank participant

Re-iterate confidentiality

**Appendix S2** Example of text included in clinic letters copied to patients

***Reconstruction***

If you choose to have a mastectomy and reconstruction, we discussed the methods of reconstruction.

The simplest option is to have a simple mastectomy and use an external prosthesis in the bra, which gives the appearance of a breast in clothes. The skin of the breast is also removed. It has the fewest risks and fastest recovery, but obviously leaves you without a breast. If you choose this option, you can have a reconstruction later (delayed reconstruction).

If you have a reconstruction at the same time as your mastectomy (an immediate reconstruction), the breast is removed, but the skin of the breast is preserved. The breast skin provides a pocket for the reconstruction and can give a more natural, realistic appearance. However, there is a higher chance of local recurrence (the tumour coming back later in the skin of the breast). Sometimes some skin must be removed, if the cancer is too close to the skin, or if the skin is too thin to be healthy.

An implant can be used under the skin to give the appearance of a breast. Implants are available in different sizes and shapes, but will always feel firmer than a normal breast and also have less droop and a less natural appearance, especially over time. The risk of an implant reconstruction not working is about 10%. If radiotherapy is required, implant reconstruction tends to deteriorate over time, with infection, capsular contracture and other complications and overall a worse result.

Autologous reconstruction (using some of your own tissue to perform a breast reconstruction) tends to give the most natural and highest quality reconstruction. However, it requires scars elsewhere on the body. We discussed using either the tissue from your back (which would also require a small implant), thigh or the tissue from your tummy, which would be sufficient in volume without implant.

We discussed all the risks and benefits of autologous reconstruction. Most people recover well without complications. Your risk of complications increases with weight and reduced fitness, which is why I recommend that if needed, you reduce weight (Body Mass Index <30) before surgery. Risks include include blood clots (2%), infection (10%), bleeding (5%), haematoma (5%), return to theatre (5%), flap failure (2%), partial flap necrosis (5%), abdominal weakness or hernia (2%), numbness (100%), asymmetry (10%).
